# Different domains of dengue research in the Philippines: A systematic review and meta-analysis of questionnaire-based studies

**DOI:** 10.1371/journal.pone.0261412

**Published:** 2021-12-20

**Authors:** Rhanye Mac Guad, Rogie Royce Carandang, Judilynn N. Solidum, Andrew W. Taylor-Robinson, Yuan Seng Wu, Yin Nwe Aung, Wah Yun Low, Maw Shin Sim, Shamala Devi Sekaran, Nornazirah Azizan

**Affiliations:** 1 Faculty of Pharmacy, Department of Pharmaceutical Life Sciences, Universiti Malaya, Kuala Lumpur, Malaysia; 2 Faculty of Medicine and Health Science, Department of Biomedical Science and Therapeutics, Universiti Malaysia Sabah, Kota Kinabalu, Malaysia; 3 Department of Community and Global Health, Graduate School of Medicine, University of Tokyo, Tokyo, Japan; 4 College of Pharmacy, University of the Philippines, Manila, Philippines; 5 School of Health, Medical & Applied Sciences, Central Queensland University, Brisbane, QLD, Australia; 6 College of Health & Human Sciences, Charles Darwin University, Casuarina, NT, Australia; 7 College of Health Sciences, Vin University, Gia Lam District, Hanoi, Vietnam; 8 Centre for Virus and Vaccine Research, School of Medical and Life Sciences, Sunway University, Selangor, Malaysia; 9 Department of Biological Sciences, School of Medical and Life Sciences, Sunway University, Selangor, Malaysia; 10 Faculty of Medicine & Health Sciences, UCSI University, Port Dickson, Negeri Sembilan, Malaysia; 11 Faculty of Medicine, University of Malaya, Kuala Lumpur, Malaysia; 12 Asia-Europe Institute, Universiti Malaya, Kuala Lumpur, Malaysia; 13 Department of Pathology and Microbiology, Faculty of Medicine and Health Science, Universiti Malaysia Sabah, Kota Kinabalu, Malaysia; US Centers for Disease Control and Prevention, Dengue Branch, PUERTO RICO

## Abstract

**Background:**

Dengue is the most rapidly spreading mosquito-borne viral disease of humans worldwide, including southeast Asia region. This review provides a comprehensive overview of questionnaire-related dengue studies conducted in the Philippines and evaluates their reliability and validity in these surveys.

**Methods:**

A review protocol constructed by a panel of experienced academic reviewers was used to formulate the methodology, research design, search strategy and selection criteria. An extensive literature search was conducted between March–June 2020 in various major electronic biomedical databases including PubMed, EMBASE, MEDLINE and ScienceDirect. A systematic review and meta-analysis (PRISMA) were selected as the preferred item reporting method.

**Results:**

Out of a total of 34 peer-reviewed dengue-related KAP studies that were identified, 15 published from 2000 to April 2020 met the inclusion criteria. Based on the meta-analysis, a poor mean score was obtained for each of knowledge (68.89), attitude (49.86) and preventive practice (64.69). Most respondents were equipped with a good knowledge of the major clinical signs of dengue. Worryingly, 95% of respondents showed several negative attitudes towards dengue prevention, claiming that this was not possible and that enacting preventive practices was not their responsibility. Interestingly, television or radio was claimed as the main source of gaining dengue information (range 50–95%). Lastly, only five articles (33.3%) piloted or pretested their questionnaire before surveying, of which three reported Cronbach’s alpha coefficient (range 0.70 to 0.90).

**Conclusion:**

This review indicates that to combat the growing public health threat of dengue to the Philippines, we need the active participation of resident communities, full engagement of healthcare personnel, promotion of awareness campaigns, and access to safe complementary and alternative medicines. Importantly, the psychometric properties of each questionnaire should be assessed rigorously.

## Introduction

Mosquito-borne pathogens, such as the causative agents of malaria, chikungunya, Zika and dengue, are major contributors to the global burden of human infectious disease [1]. In particular, the geographical distribution of dengue virus has increased alarmingly in recent decades to become a worldwide public health concern [2]. Currently, this flavivirus is reported in around 130 countries, with up to 400 million new cases of clinical infection recorded annually [3]. It is hyperendemic in southeast Asian countries, including Cambodia [4], Malaysia [5], Thailand [6], Bhutan [7], Brunei [8], Indonesia [9], Myanmar [10], Vietnam [11] and the Philippines [2,12]. The World Health Organization (WHO) projects that in excess of 2.5 billion people live in dengue-endemic areas, a significant contributing factor to an estimated annual death toll of 25,000 [13]. Nonetheless, the possibility of unapparent and under-reported infections should be recognized, not only due to accelerating geographical spread but also passive case detection; for instance, failure to detect persons with dengue who do not seek health care or to report all symptomatic dengue patients [14,15].

In common with many other tropical countries the risk level of dengue in the Philippines is considered as frequent or continuous due to regular outbreaks or ongoing transmission [16]. This is affected by several factors such as seasonal meteorological patterns (mean temperature, average relative humidity, and total rainfall) [17], increased urbanisation and volume of international air travel [18] that has led to an increase in the viability/reproduction/range of *Aedes* vector mosquitoes. Despite the fact that the first published report of a dengue epidemic in southeast Asia is from 1954, dengue outbreaks in the Philippines were documented in hospital records as early as 1926 [19]. During the years 2000–2011 all 17 administrative regions of the Philippines reported increased incidence of dengue, especially in the most populated urban areas, with all four virus serotypes co-circulating and exhibiting temporal and spatial variation. It is estimated that 80% of dengue-related deaths occurred in individuals ≤ 20 years old, with the highest number of cases in children between 5–14 years of age [18]. Most recently, in 2019 the Philippines Department of Health (DOH) issued a dengue alert in several regions due to a drastically elevated (85%) clinical case load over a six-month period [20,21]. Although the overall incidence of dengue in the Philippines has risen more than eight-fold between 2000 to 2019, this could be partly due to the altered reporting and recording system of dengue cases employed by the WHO and the Philippines DOH.

Several measures to prevent or control mosquito behaviour and breeding have been recommended in order to combat the spread of dengue virus. These actions include: personal protection from mosquito bites; provision of public engagement activities to educate local communities to improve household participation rates against the mosquito vector; emergency use of insecticides in outbreaks to achieve reactive vector control; and rolling out a range of local government-led proactive mosquito control and surveillance initiatives [22,23]. Similarly, the Philippines DOH has developed national programmes for dengue prevention and control, comprising surveillance, case management and diagnosis, integrated vector management, outbreak response, health promotion and advocacy, and research. Moreover, the DOH has implemented a so-called 4S strategy (Search and destroy, Seek early consultation, Self-protection measures, Say yes to fogging only during outbreaks) to strengthen the policy’s effectiveness [24]. Both the Philippines Integrated Disease Surveillance and Response and the Department of Virology of the Research Institute for Tropical Medicine took part in this programme, particularly in regard to surveillance.

For questionnaire-based research, different behaviours and perceptions are used to measure social aspects of dengue in the Philippines, such as knowledge, attitude and preventive practices (KAP), dengue vaccine acceptance, the health belief model (HBM) association with dengue, and complementary and alternative medicines (CAM) to treat dengue. Far-reaching conclusions have been drawn from questionnaire surveys conducted in other endemic countries, providing a useful guide to decision makers in setting health policy priorities [25], assessing dissemination, application and cost-effectiveness of current guidelines, and closing important gaps in our knowledge of patterns of dengue transmission [26]. Studies have suggested that a combination of multidisciplinary and bottom-up approaches is more likely to be successful and sustainable way to combat dengue [27]. Prevention and control should be promoted in school and university curricula, as should the crucial role of healthcare volunteers in implementing effective social networks to raise dengue awareness of householders that may influence their attitudes and behaviour towards dengue [28]. Despite this, there has been a limited number of questionnaire-based studies in the Philippines compared to neighbouring countries, such as Malaysia, Thailand, and Indonesia.

Furthermore, the collective scopes have not been discussed previously in the context of researching a pattern for guidance. In addition, the accuracy of findings from questionnaire-based studies is a matter of concern, as the accuracy of results depends largely on the reliability of the questionnaires used in the survey [29]. A comprehensive review of questionnaire-based dengue-related studies is required to highlight the findings from all relevant previously published work on the behavioural and practice aspects related to dengue prevention, to assess the validity and reliability of questionnaires used in such research, as well as to draw broad conclusions. Thus, this systematic review and meta-analysis aims to summarize existing questionnaire-based studies conducted in the Philippines, which may help to improve survey design relating to different domains on the behavioural and practice aspects related to dengue infection. In addition, it highlights future research needs and serves as a valuable reference for policymaking or health interventions focusing on the Filipino population.

## Methodology

### Study design

The research protocol is in line with recommendations outlined in the Preferred Reporting Items for Systematic Reviews and Meta-Analysis (PRISMA) guidelines [13] (S1 Appendix).

#### Search strategy

**Fig 1:**
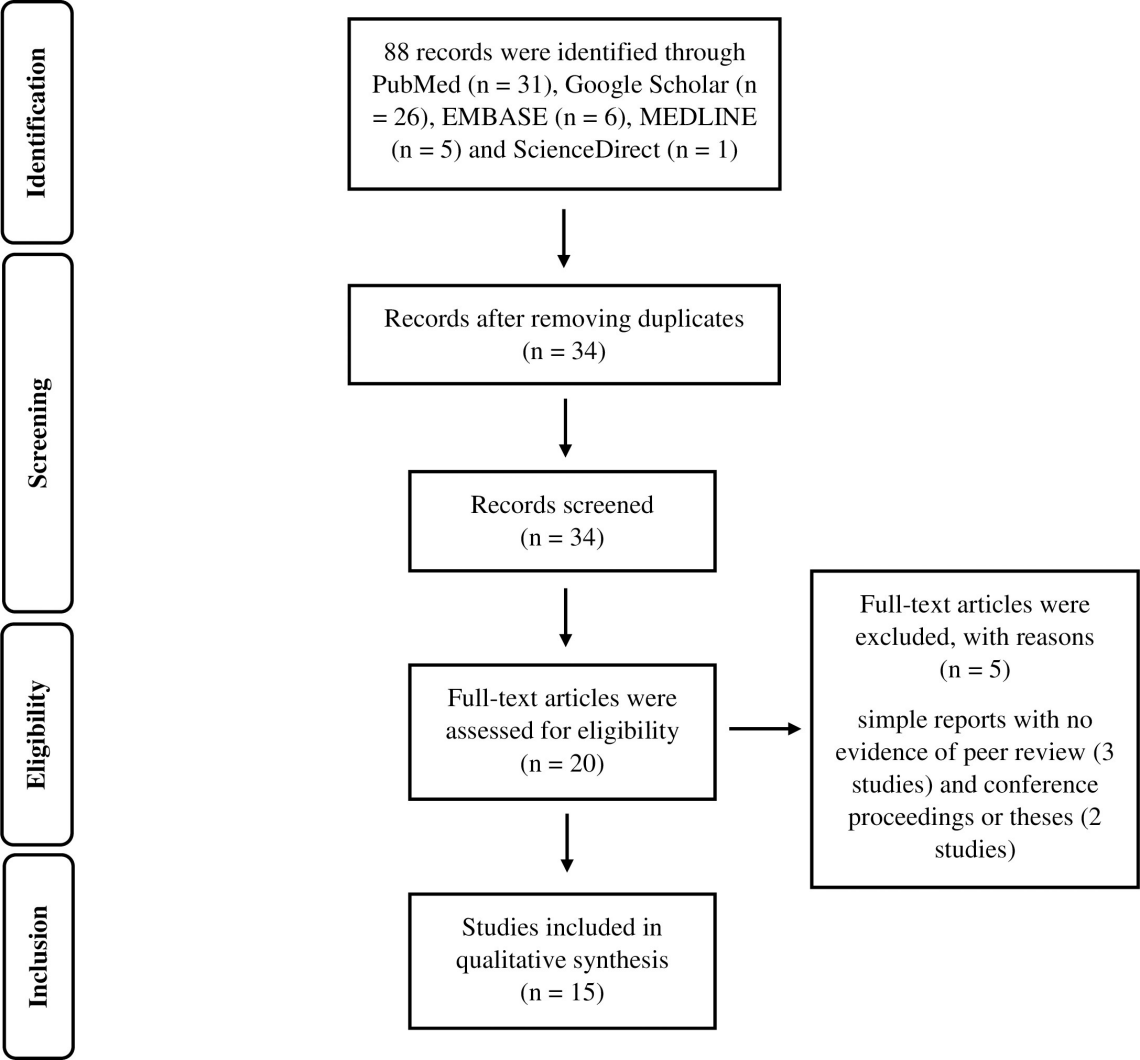
PRISMA flowchart of the literature search.

The methodology, research design, search strategy and selection criteria were based on the review protocol (S2 Appendix) developed by the team of researchers who comprise experts in public health, infectious diseases and clinical medicine. An extensive literature search was conducted during March–June 2020 using various major electronic biomedical databases, such as PubMed, Goggle Scholar, EMBASE, MEDLINE and ScienceDirect. A checklist of preferred reporting items for systematic reviews and meta-analysis (PRISMA) [30] was used to present the flow of research strategy, consisting of selection, including and excluding the relevant articles, as shown in Fig 1.

#### Screening and eligibility

All articles in English that were collected, compiled and eventually included in this review, reported a peer-reviewed dengue questionnaire-related research study conducted in the Philippines and published between January 2000 –April 2020. Moreover, any article considered as part of the review was cross-checked through references and in-text citations to ensure the inclusion of all relevant articles. In this review, we offered no restrictions on the type of participants included in the study; community residents, university students, in-patients, parents, and caregivers were included. For the intervention/exposure, all questionnaire-related dengue studies conducted in the Philippines were included. We did not have any comparison group in this review. Regarding the outcomes, we summarized the main findings that reported dengue-related knowledge, attitude, and practices.

A non-questionnaire-based study (13 studies), simple reports with no evidence of peer review (three studies) and conference proceedings or theses (three studies) were excluded due to lack of information for data extraction and/or evidence of peer review. The relevance of each article was determined using individual keywords or a string combining ‘dengue’, ‘questionnaire’ and ‘Philippines’. Additionally, the Boolean operators “AND”, “OR” and “NOT” were used to link categories of keywords, thereby aiming to increase sensitivity and specificity of the query. No limits by sex, age and ethnicity of study participants as well as language of the articles were imposed. Similar keyword(s) were applied to all electronic databases to search for articles.

#### Data extraction and management

The decision whether or not to include each article was made after reaching a consensus among the research team following group discussion between members via email. A total of 34 articles were retrieved electronically, with further papers that were not open access being acquired by emailing the paper’s corresponding author. After removing duplicate publications 14 articles were identified as either irrelevant or to not fulfil the abovementioned criteria, and hence each was excluded.

#### Risk of bias assessment

The remaining 20 articles were assessed further during the first round of review, undertaken by six expert reviewers (public health authorities and infectious diseases specialists) based on titles and abstracts, from which five articles were eventually excluded for not being a questionnaire-related study (e.g. a workshop protocol on dengue prevention and control or fieldwork on breeding sites of *Aedes* species mosquito). The second round of review was performed by three expert reviewers to ensure that based on the selection criteria only relevant articles were included in the final selection; no further papers were excluded. All papers fulfilling the inclusion criteria were critically appraised based on the eight critical appraisal tools of the critical appraisal skills programme (CASP) Checklist [31] (S3 & S4 Appendices). Therefore, this review contained a total of 15 articles, as indicated in Table 1. In this systematic review, 15 papers related with knowledge, attitude and practice on dengue study in the Philippine were included. However, as the number of studies fewer than 10 in the meta-analysis, not all these papers recorded the same effect size. Therefore, an assessment of publication bias using graphical methods (e.g. funnel plot asymmetry) or statistical methods (e.g. Egger’s test) was not possible.

**Table 1:** List of published questionnaires-based dengue studies in the Philippines, 2004–2020.

Reference	Location of study	Respondents (n)	Sampling method and data collection method	Main findings
Rufo & Amparado [24] *No evidence of pilot*	City of Naga, Cebu	Community residents (400)	Quota sampling through self-administration	• High fever recognized as a symptom of dengue • Preventive practices included indiscriminate fogging, cleaning water storage vessels by scrubbing and cleaning roof gutters once a week • Significant relationship between respondents’ highest educational attainment with search and destroy control measures
Kwon & Crizaldo [32] *No evidence of pilot*	Dolores Barangay, Taytay, Rizal	Community residents (48)	Purposive random sampling	• 56.3%, 95.8% and 50% of participants demonstrated knowledge, good attitude and preventive practices, respectively • Television was the main source of information on dengue (93.8%)
Pinton & Demayo. [33] *No evidence of pilot*	Lugait, Misamis Oriental	Community residents (300)	Random sampling through self-administration	• Major sources of information were mass media, health centres, and neighbours
Lubos & Lubos. [34] *No evidence of pilot*	Malaybalay City, Bukidnon	Mothers (280)	Random sampling through self-administration	• Knowledge about other symptoms of dengue was low among participants • Participants demonstrated a good attitude towards preventive practices
Mahilum et al. [35] *No evidence of pilot*	Cebu City	Community residents (489)	Interview	• 68.7% of participants were aware that dengue is transmitted by mosquitoes, but only 4.3% recognized dengue virus as the cause of disease
Lozano et al. [36] *No evidence of pilot*	Cebu City	Community residents (50)	Random sampling through self-administration	• No association between demographic variables and either knowledge or preventive practices • No correlation between knowledge of dengue and preventive practices
Abvia et al. [37] *Pretested/piloted*	Barangay Kauswagan, Cagayan de Oro City	Community residents	Purposive sampling through self-administration	• Main sources of information included mass media, healthcare brochures and home visit • Preventive practices were using mosquito nets and avoiding, and/or reducing outdoor activities
Herbuela et al. [38] *Cronbach’s α coefficient = 0*.*75*, *0*.*76*, *and 0*.*76*	Metro Manila	In-patients (250)	Case control through semi-structured interview	• Dengue patients demonstrated significantly lower mean scores in the practice domain compared to controls (*p* < 0.001) • Being in senior high school, having experienced hospital and having had a rash were predictors of knowledge and good attitude in paediatric patients • No correlation between each of knowledge and attitude with preventive practices
Lennon [39] *No evidence of pilot*	Foundation University, College of Education, Dumaguete City	University students (43)	Purposive sampling through open-ended semi-structured questionnaire	• Most important measures for mosquito larval control included cleaning residences and their surroundings, elimination of stagnant water, not exposing open cans and use of insecticide spray • Perceived barriers to achieve mosquito larval control were apathy, laziness and lack of time
Yboa & Labrague [40] *Cronbach’s α coefficient = 0*.*90*	Samar Province	Community residents (646)	Convenience sampling through self-administration	• 61.45% demonstrated good knowledge • > half of respondents used electric fans, mosquito coils and bed nets as preventive measures No correlation between knowledge and preventive practices (*p* = 0.75) • Television/radio was main source of information
Carandang CM & Resurreccion [41]	Philippine Children’s Medical Center–Outpatient Department, Quezon City	Parent and caregivers (139)	Purposive sampling through self-administration	• Dengue vaccine acceptance among participants was 81.3% • Educational attainment, employment status and monthly income were significantly associated with vaccine acceptance
Carandang RR et al. [42] *No evidence of pilot*	Sta. Cruz, Laguna	Community residents (32)	Random sampling through assisted interview	• Rash attributed as the prominent sign and symptom (88%) followed by fever, headache, muscle pain, abdominal pain and joint pain
Reyes et al. [43] *No evidence of pilot*	Metro Manila	Caregivers (202)	Purposive sampling through focus group discussion	• Household size, knowledge regarding dengue and attitude towards vaccination were significantly associated with willingness for vaccination.
Palanca-Tan [44] *No evidence of pilot*	Quezon City, Manila	Community residents (205)	Interview	• Willingness to pay for vaccination ranged between a mean price of USD 27–32
De Guzman et al. [45] *No evidence of pilot*	Anda Island, Mt. Colisao and Mt. Balungao, Pangasinan	Community residents (82)	Interview	• High fidelity levels (FL) values and corrected major use agreements (cMUA) of at least 35% were obtained for cardinal symptoms of dengue relating to bleeding episodes, while low cMUAs (2–4%) were obtained for symptoms during the recovery phase • High FL values were obtained for symptoms observed during the febrile phase.

### Statistical analysis

We conducted quantitative synthesis to derive meta-estimates of knowledge, perception and attitude of the study population and qualitative synthesis to describe the study population, study design, sampling methodology and outcomes presented in the paper. For each study, primary outcome (knowledge, attitude and practice score) and secondary outcome (percentage of population with good knowledge, acceptable attitude, and practice) were extracted. Knowledge, attitude and practice score were standardized to cent percent and pooled estimates are presented as mean and 95% confidence interval. Prevalence of population with good knowledge, acceptable attitude and practice were also identified, meta-analysed and presented also as mean and 95% confidence interval. Forest plots were used to display pooled estimates. Heterogeneity was tested using likelihood ratio test. Analyses were performed using STATA 16 statistical software. For meta-analysis interpretation, based on previous studies [27,40] the cut-off values used for standardized knowledge scores were as follows: < 64, poor; 64–80, good; > 80, very good.

## Results

### Awareness and knowledge of dengue infection

**Fig 2:**
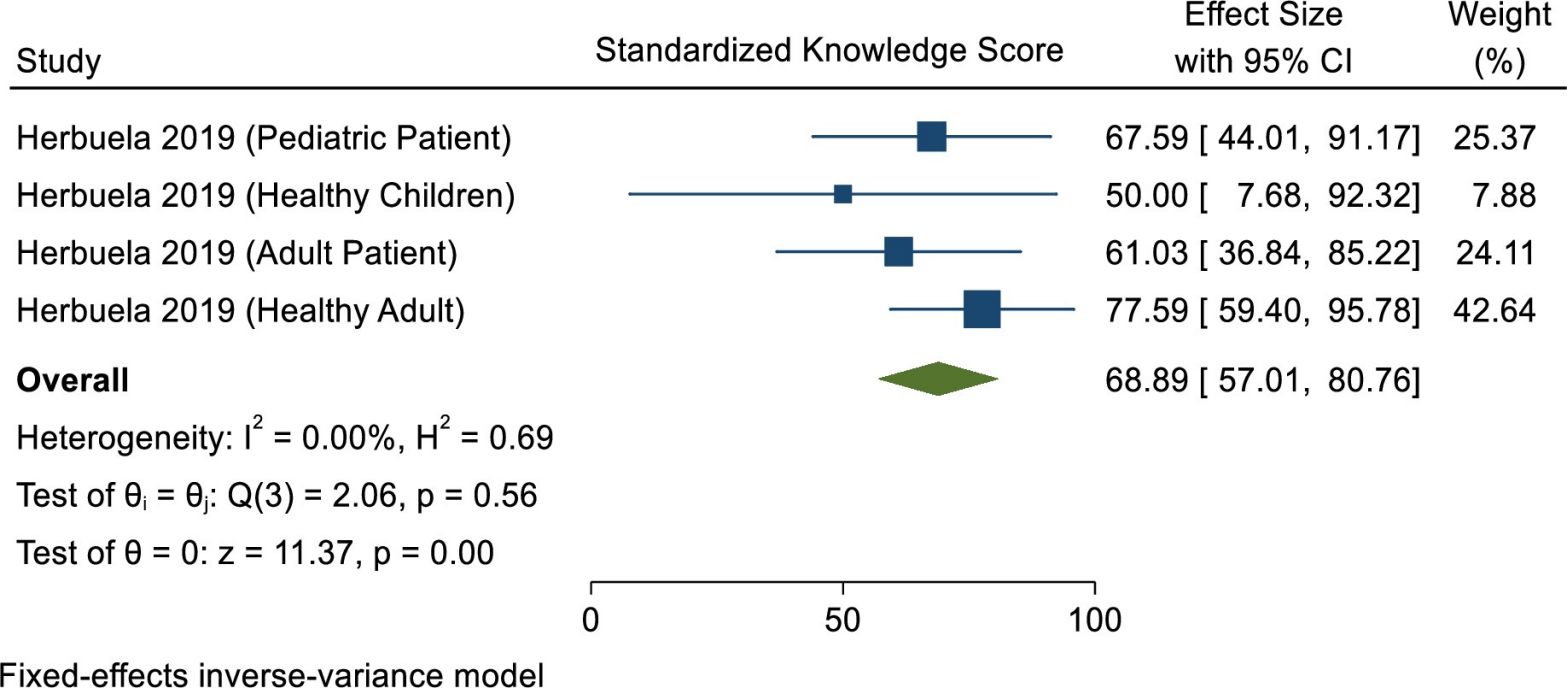
Meta-analysis of dengue knowledge scores in the Philippines.

Based on percentage scale, the mean knowledge score was 68.89 (Fig 2). The current systematic review shows that most respondents (95%) held several erroneous beliefs: that (1) dengue transmission cannot be prevented; (2) elimination of larval breeding sites is the responsibility solely of public health staff and healthcare volunteers; (3) eliminating such sites is very complicated, poor use of public funds and a waste of time; (4) insecticide fogging is sufficient to prevent mosquitoes; (5) individuals who have experienced dengue infection once cannot be infected subsequently; and (6) a fit and healthy person will not get dengue infection [32,35].

**Fig 3:**
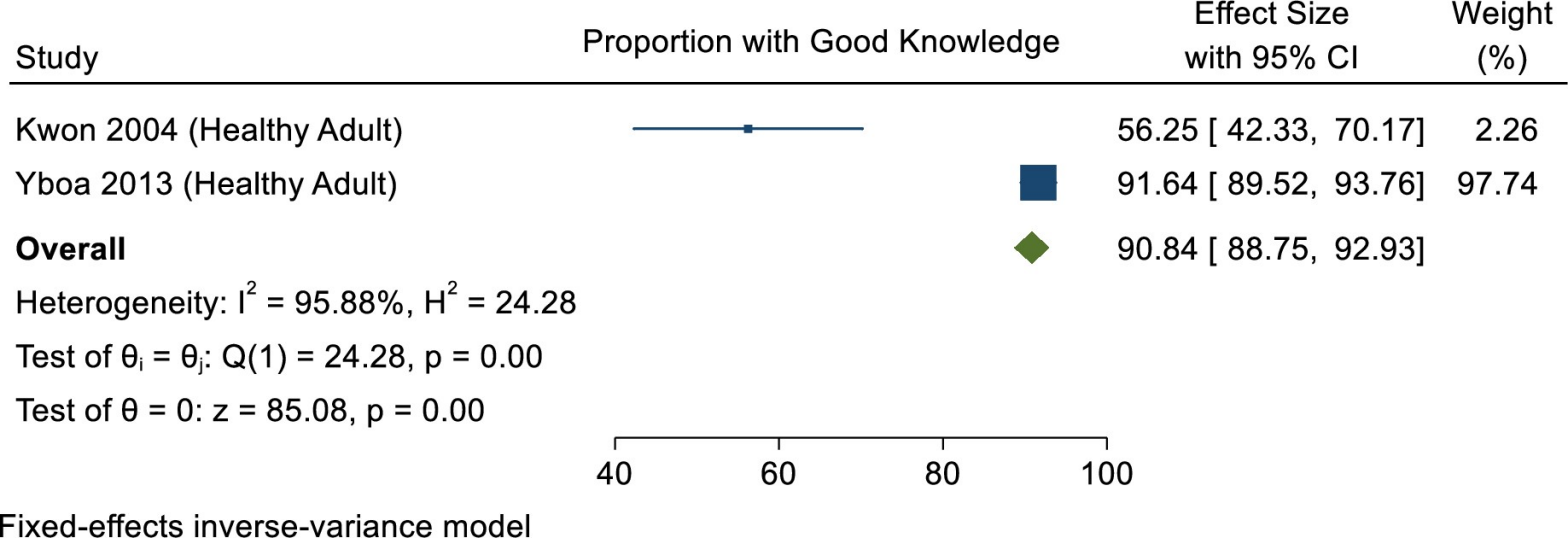
Meta-analysis of proportion of people with good dengue knowledge in the Philippines.

In 2004, Kwon & Crizaldo [32] reported that more than half of participants (56.3%) had moderate knowledge of dengue, whereas in 2013 Yboa & Labrague [40] reported higher knowledge of dengue (91.6%) among rural residents in Samar Province, Philippines. This is despite the fact that the two studies employed different sampling techniques: the former used purposive random sampling [32], whereas the latter used convenience sampling [40] (Fig 3). In terms of recognition of symptoms of dengue infection, most respondents answered correctly that fever is the major clinical feature of uncomplicated dengue [24,32–34] affecting infants, young children and adults [33]. Other symptoms claimed by respondents include chills, headache, pain upon eye movement, lower back ache, stomach ache, skin rashes, vomiting, bleeding of the nose and gums, muscle pain and diarrhoea [32,33,35,36].

Interestingly, most respondents answered correctly questions assessing their knowledge of dengue transmission. For instance, they identified that the dengue virus is transmitted to humans through the bite of an infectious female *Aedes aegypti*: 89.6%, [32]; 56.07%, [34]. Furthermore, the majority of respondents recognized that *Aedes* mosquitoes bite during daytime: 66.7%, [32]; 28.85–46%, [36]; 17–37.67%, [33]. However, an incorrect perception of biting time at night has also been reported; 64%, [34]. Up to 95.8% of survey participants correctly identified stagnant water collected in discarded tyres, trash cans and flowerpots as providing good breeding sites for mosquitoes [32,36]. More than 50% of respondents acknowledged that not all mosquitoes carry dengue, flies and ticks do not transmit the virus and that disease may be contracted through transfusion of infected blood [34], and also that heavy rainfall provides conditions favourable to rising numbers of mosquitoes responsible for dengue [33], due to formation of larval breeding sites [32]. Additionally, around 50% of respondents claimed that combating dengue vector mosquitoes is the only way to control infection [32], or that sleeping under a mosquito net prevents infection [37]. Only 25% of respondents realised either the possibility of contracting dengue if a family member had been infected with the virus or that the rainy season (June–February) is, historically at least, the only epidemic period for dengue infection in the Philippines [32].

The knowledge of survey participants regarding an individual’s risk of dengue infection was unsatisfactory as 23% thought that a fit and healthy person could not be infected more than once in a lifetime. Moreover, only 12.5% of respondents strongly agreed that it is possible to recover completely from infection [32]. A study by Herbuela et al. (2019) [38] demonstrated that knowledge of dengue is not always directly proportional to educational attainment; for instance, paediatric patients in senior high school knew more about dengue compared to those who were in college who had experienced dengue for the first time.

### Attitude towards dengue infection

**Fig 4:**
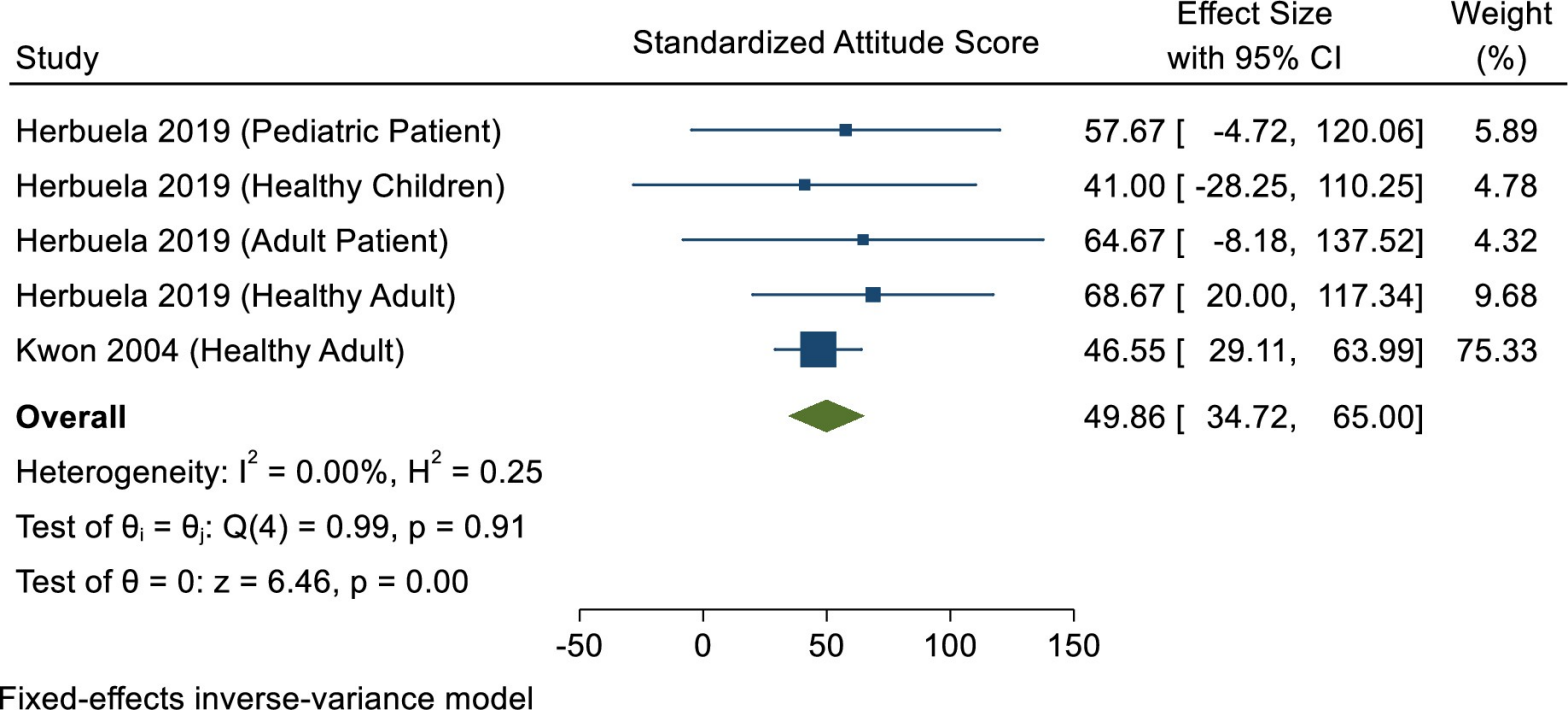
Meta-analysis of attitude scores among Philippines communities.

After standardising to percentage scale, the overall attitude score was 49.86, reflecting a poor attitude among Philippines populations towards dengue (Fig 4).

It is of interest to note that those patients who were high school seniors or who experienced a longer stay in hospital tended to have a better attitude towards dengue. However, this association decreased as the patients aged [38]. Other positive attitudes of Filipino communities when infected with dengue were reported [36]. These included consulting a physician, taking plenty of rest, drinking copious water when affected by the disease, and seeking herbal medicine (mangagaw, tawa-tawa or gatas-gatas). Drinking apple tonic, installing residential door and window screens, sleeping under mosquito nets, and burning mosquito coils and dried leaves were each also mentioned as a method used to prevent dengue.

Furthermore, two studies have demonstrated a good attitude towards dengue among mothers of young children in Malaybalay, the capital city of the province of Bukidnon. They believed that dengue is a serious disease (60%); it cannot be treated at home (92.5%); it is preventable (70.7%); it can be prevented by controlling breeding sites of mosquitoes (69.6%); government is not solely responsible for control (71.7%); and control requires active community participation (95%) [32,34]. Lennon (2004) reported that students from Dumaguete, a city on Negros Island in the southern Philippines, showed a positive attitude towards dengue control and prevention as they practised the following: (1) cleaning inside their house and its immediate surroundings; (2) eliminating collection of stagnant water by keeping opened cans and other vessels upturned or in a suitable place; and (3) applying insecticide spray, all of which are measures of mosquito larvae control [39]. However, Lennon (2004) also mentioned that lack of knowledge and correct behaviour (characterized as ignorance, apathy, laziness, perceived lack of time and/or lack of cooperation) among students could manifest in poor attitudes towards combatting dengue.

### Preventive practice towards dengue infection

**Fig 5:**
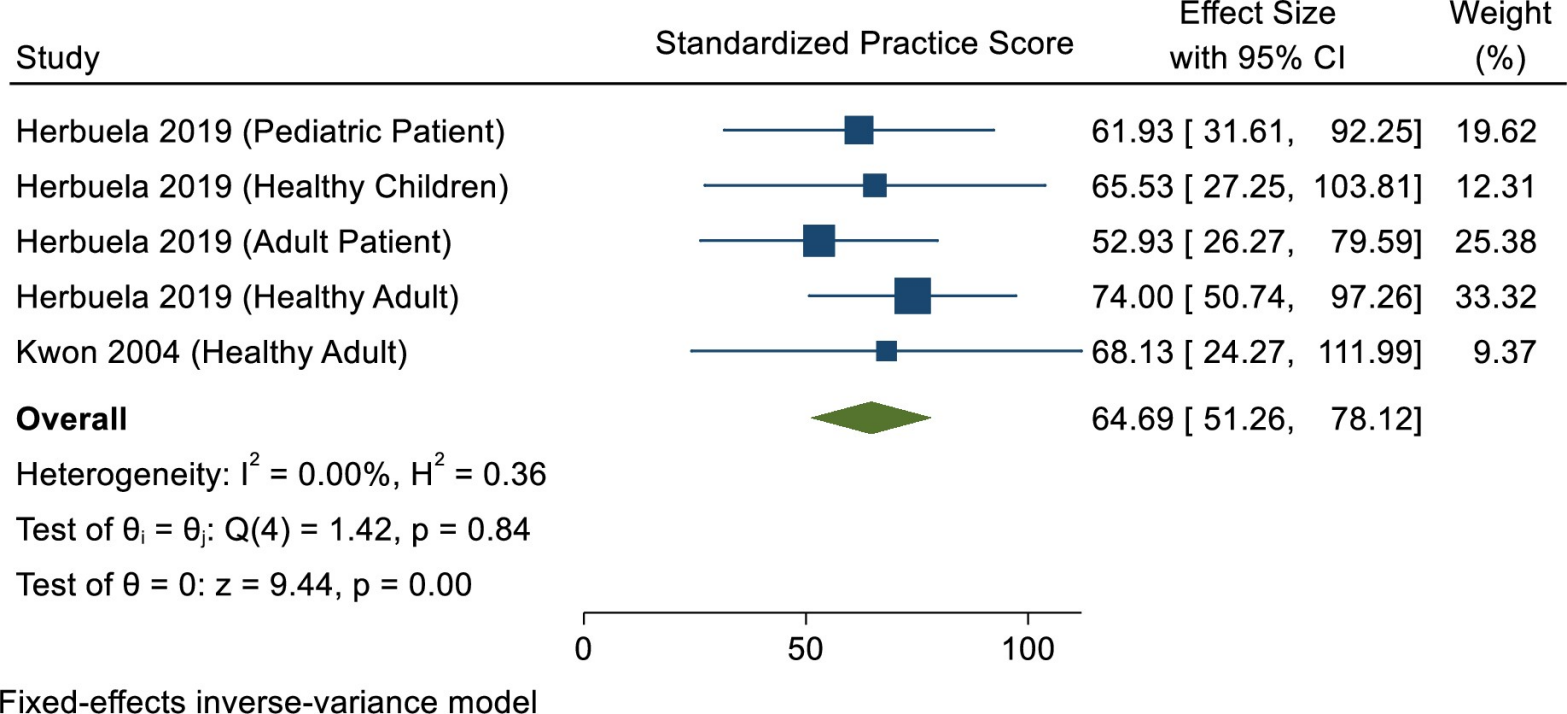
Meta-analysis of dengue practice score in the Philippines.

After standardising to percentage scale, the overall practice score was 64.69%, indicating that preventive practice towards dengue among Filipino populations is acceptable regardless of a poor attitude score (Fig 5). Based on previous studies, the most preferred options for preventive practice were “search and destroy mosquito breeding sites” including covering water storage containers after use (> 90%), examining toilet cisterns for mosquito larvae (88%), regular disposal of refuse into garbage bins (> 80%) [33,35]. Progressively less popular options included using mosquito nets/mosquito coils in the house (77%), checking and cleaning roof gutters during the rainy season (69%), and insecticide fogging (67%) [32,35,40,41].

Further results showed that the best self-protection method was covering water storage containers immediately after use (92%) [32,33,40], screening of windows (88%), use of mosquito bed nets (92%) or using an electric fan [33,35,40,41]. Some survey respondents (63%) reported that they had used professional pest control in an attempt to prevent dengue infection [40,41]. Other preventive measures that have been practised by respondents are: (1) traditional fogging to disperse mosquitoes, especially during the afternoon; (2) wearing long-sleeved shirts and trousers, especially by small children. A recent study by Rufo & Amparado (2017) indicated the practices of scrubbing water storage vessels and cleaning roof guttering at least once a week or a preference for fogging to be important means of dengue management practice [24]. Additionally, Herbuela et al. (2019) revealed that mosquito larvae-eating fish, screen windows and dengue vaccination were each identified as a protective factor against dengue infection, of which the biological control method of using larvivorous fish was the strongest factor in the model with an adjusted odds ratio (AOR) of 8.69 (95% CI: 3.67–20.57, *p* ≤ 0.001) [38].

In contrast, a study in Lugait, a municipality in the province of Misamis Oriental, identified several negative practices performed by resident communities [33]. These included: (1) leaving water storage containers uncovered for more than a week inside the house (57.67%); (2) overwatering of flower vases and potted plants (21.67%); (3) the presence in the neighbourhood of plants such as bananas in which mosquitoes are known to shelter (65.33%); (4) discarding tyres, cans, bottles and other containers in which water may collect (43.33%); (5) rivers, ponds and puddles of water that form after raining (26.00%); (6) coconut shells (23.67%); (7) no proper drainage (13.67%); and (8) dirty surroundings (9.67%).

### Treatment-seeking behaviour

In terms of treatment-seeking behaviour, most respondents associated fever as being the principal manifestation of dengue infection (86.5%) [24]. Interestingly, parents would preferentially choose to bring a child with fever to a district hospital (54.75%) rather than to a rural health unit (44.75%), private clinic (40.25%), tertiary hospital (27.50%) or quack doctor (7.5%).

### Sociodemographic variables and KAP regarding dengue infection

Based on this meta-analysis, a significant positive correlation between knowledge and attitude domains was observed among paediatric patients with confirmed dengue infection, although this is not statistically strong (Spearman’s rank correlation coefficient, Rs = 0.2). However, Herbuela et al. (2019) reported that neither the knowledge nor the attitude of dengue patients correlated with their practices [38]. Other studies have also reported an insignificant association between sociodemographic variables, knowledge, attitude or practice regarding dengue among communities in the Philippines [24,32,36,40]. Sociodemographic and economic data collected include, for example, age, sex, education, migration background and ethnicity, religious affiliation, marital status, household, employment, and income.

### Sources of information on dengue infection

It is noteworthy that most of the articles analysed reported the main source of information on dengue infection being provided by television (ranging from 49.7% to 93.8%) [32,33,37,40] and radio (73.37%) [33,37,40]. Yet, respondents gained information from a variety of other sources: for instance, via health workers (80.33%) [32], (5.11%) [40]; schools (34.00%) [33]; internet (9.67%) [33]; posters (3.33%) [33]; and by speaking with neighbours and/or friends (4.20%) [32,33,37].

### Dengue vaccination

In a hospital-based cross-sectional study, the acceptance rate of dengue vaccination was 81.3% (113 out of 139) among parents and caregivers. Completion of secondary or tertiary education (AOR = 0.22, 95% CI: 0.01–4.1, *p* < 0.0001) and lower income group (AOR = 2.8, 95% CI: 0.06–5.1, *p* < 0.0001) were the independent factors associated with dengue vaccine acceptance [40]. On the other hand, a community-based survey revealed that 95.5% (193 out of 202) of primary caregivers accepted dengue vaccination, a very high rate [42].

Based on this meta-analysis, good attitude towards vaccination (AOR = 10.62, 90% CI: 1.73–26.28) and large household size (AOR = 9.63, 90% CI: 2.04–45.38) were each positively associated with vaccine acceptance within a community. In contrast, good knowledge of dengue (AOR = 0.10, 90% CI: 0.03–0.74) and age of 44 years or more (AOR = 0.14, 90% CI: 0.03–0.61) were two factors that negatively influenced acceptance rate [43].

For the aspect of willingness to pay (WTP) for a single dengue vaccine and the household demand function for dengue vaccines, Palanca-Tan (2008) reported that the mean WTP for a vaccine ranged from USD 27–32, and the household demand averaged two persons per household [44]. For lower income groups with less capacity to pay, a mass vaccination campaign programme was suggested instead, through which at least part of the financial costs is covered.

### Complementary and alternative dengue prevention

Indigenous communities in the province of Pangasinan, located on the island of Luzon, use *Euphorbia hirta*, locally known as tawa-tawa, as a Filipino tradition for dengue [45]. The most widely used treatments are decoctions of the leaves and bark. The plant extract was reported to be effective as a symptomatic CAM for dengue in the initial, febrile and recovery stages, as well as for supportive therapy [45].

### Reliability and validity of questionnaire

Out of 15 articles reviewed, 5 had piloted or pretested the questionnaire [24,37,38,42,44] before surveying. Three articles that were adapted [34,38,40] and 8 articles containing a new questionnaire [32,33,35,36,39,42,43,45] were considered as having a high risk of bias on the questionnaire due to lack of evidence on reliability and validity.

## Discussion

This systematic review provides the first description and insight into questionnaire-based studies conducted in different dengue-endemic communities in the Philippines, where an upward trend of dengue cases has been reported for more than a decade [12]. Filipinos prefer a healthcare facility that provides a higher level of medical attention than those offering basic services despite the availability of the latter in the locality of respondents. Television and radio play an important role in delivering dengue information to resident communities. Although a high vaccination acceptance rate was reported among community residents, this needs to be re-assessed due to the ‘Dengvaxia’ dengue vaccine controversy. It was found that the majority of respondents have an inadequate KAP level regarding dengue, which is associated with several factors.

The Philippines, like many other countries in the tropics, is plagued by dengue [16,46]. For decades vector surveillance and control measures have remained the mainstay of dengue control and prevention programmes. There is a pressing need for these to be effective as dengue has no cure and patients are subjected only to symptomatic management after becoming infected [2]. Moreover, the only vaccine currently available, Dengvaxia®, has variable safety and efficacy by age and serostatus such that its licensure has proved controversial [47]. In fact, human living practices play a crucial role in maintaining dengue virus transmission via *Ae*. *aegypti* carriage by providing a suitable breeding environment and ready source of blood meal for this peridomestic dengue vector. Therefore, here we have focused on questionnaire-based studies in relation to different domains of human behaviour towards dengue, such as KAP, sources of information, preventive treatments, HBM and CAM. In addition, this review also analysed the reliability and validity of survey questionnaires used in the included articles. The findings reported herein stress that the development of a proactive dengue control programme should be prioritized in order to protect the health of all layers of Filipino society, especially those located communities in highly endemic areas.

In this systematic review of the population of the Philippines, cut-off values were based on a 100-point scale (i.e. for instance, an 80.00% score is considered as good). Overall, the respondents achieved 68.89%, 49.85% and 64.69% scores for knowledge, attitude and preventive practices towards dengue, respectively. This KAP score revealed that more than half of the entire cohort had adequate knowledge regarding dengue, specifically of dengue infection per se and of its signs and symptoms. The level of knowledge among Filipinos revealed here is lower compared to studies conducted in Malaysia (more than 90%) [48] and Laos (70.9%) [49]. In comparison, 50% of the rural population in Kancheepuram district of Tamil Nadu, India [50] and the population of the earthquake- and tsunami-affected area of Aceh Indonesia [51] were knowledgeable about dengue symptoms. These findings shows that communities living in regions where the fatality rate from dengue is high have less knowledge, perhaps placing them at greater risk. Hence, increased mortality from dengue appears to be correlated with ignorance of its virus aetiology, vector transmission and disease symptoms, thereby reiterating the paramount importance of public health education programmes. Interestingly, a study conducted in Nepal reported corroborative findings that compared to participants resident in the lowlands a significantly lower proportion of those living in highland areas, which experience low dengue fatality rates, were able to correctly identify typical symptoms of dengue [28]. Similar to the observations made in the Philippines such differences may be attributed to intensified education and awareness campaigns in highly endemic areas leading to an increased level of knowledge. Due to the incrementally expanding distribution of *Aedes* mosquitoes as a direct result of climate change, future dengue awareness campaigns should target communities in both endemic and potentially endemic areas, not only in the Philippines but elsewhere in tropical and subtropical zones [52]. Among Filipino communities in areas of high endemicity for dengue, public health engagement should focus on those identified factors associated with attainment of knowledge. Plausibly, the meta-analysis also found an inverse association between level of education and knowledge of dengue, suggesting that a better understanding and comprehension of information on dengue does not necessarily depend on the level of education which a person has reached. It is recommended that in order to raise knowledge of dengue, public health campaign materials should be piloted and evaluated routinely with community members of all educational backgrounds, as well as training of personnel to deliver educational information effectively to address knowledge gaps regarding dengue in the community.

The current meta-analysis reveals a poor attitude (49.85%) and preventive practice (64.69%) towards dengue among communities in the Philippines. Approaching half of an urban community (43.80%) [32] held an erroneous belief that chemical fogging by the local government authority is adequate to reduce dengue transmission compared to only around a third reported for a similar Malaysian urban population (31.8%) [53]. In fact, existing policies should revisit the implementation of insecticide use, such as fogging periodically instead of sporadically and its deployment as an adjunct vector control method. A reduction in mosquito and larval density after fogging as measured by a drop in mean ovitrap index value from 71.67% to 69.42% has been reported [54]. However, sole dependency on preventive fogging may lead to the emergence of insecticide resistance [55]. Furthermore, of the included articles, two studies showed that some respondents believed that eliminating breeding sites is the exclusive responsibility of public health staff and healthcare volunteers (52.10% and 28.30%, respectively) [32,34]. These values are comparable with those reported by a previous study in an Indian population (49.00%) [56]. This attitude needs to change because achieving a reduction of the vector population and prevention of virus transmission requires the active participation of affected communities. In order to combat mosquito breeding, all residents should take personal responsibility to regularly clean their housing and immediate surroundings. Nonetheless, local government authorities should spearhead this effort as studies have reported that search and destroy practices require trained personnel to have good knowledge and skills to be able to remove *Aedes* breeding sites more effectively [2].

The negative behaviour among university students towards dengue prevention [38,39] could be explained by using the health belief model, conceivably due to weak confidence in the effectiveness of the proposed measures to control mosquitoes and thereby to prevent dengue transmission. A perception of reduced benefits coupled with elevated barriers may result in a lesser possibility of change, as reported in a Malaysian population [57]. Self-efficacy is another HBM construct that, in addition to the perceived threat of dengue and other parameters, encourages an individual to implement preventive practices. Not surprisingly, university students might have a low self-efficacy or confidence in doing something with which they are unfamiliar, which could have led to their low interest to carry out mosquito control tasks. In this context, although they have a higher level of education, and therefore may have the ability to understand various information on dengue [58], they may not have the self-confidence to perform regular, comprehensive environmental clean-up tasks, as claimed by studies conducted in Malaysia [59] and Pakistan [60]. Hence, it is important to identify the trigger for a person’s motivation to contribute to vector control initiatives. This is unlikely to be the same for different people or groups within a community where there will be several cultural and social factors at play. We suggest that a public health campaign should incorporate guidance on how to conduct steps of an environmental action plan for dengue control. Furthermore, this should be based on an HBM construct specific for the Filipino population in order to increase their self-efficacy and behaviours regarding mosquito control.

Pre- and post-educational intervention in Malaysia achieved via public health campaigns and further disseminated by discussion among students revealed that educational intervention was effective in generating awareness of dengue (mean scores for pre- and post-intervention were 10 ± 2.46 vs 12.61 ± 0.17, 8.82 ± 1.35 vs 9.01 ± 1.09 and 6.92 ± 2.5 vs 7.11 ± 2.49 for knowledge, attitude and practice, respectively) [61,62]. Educational intervention should include promotion of skills development that may help to reduce the perception among students of time as a limitation to performing mosquito control activities. Other studies have highlighted additional barriers to the effectiveness of public health campaigns, including not being conducted on a routine basis [63] and initiatives being driven from the top down, thus creating resistance from community residents to participate in interventions [64].

### Treatment-seeking behaviour

Most respondents claimed that fever is associated with dengue, prompting them to attend the nearest healthcare facility to seek treatment. This finding is in accordance with HBM, whereby self-regulation emphasizes that people have or can develop autonomy, self-control, self-direction and self-discipline due to the assumption that all behaviours are motivated by the desire to achieve goals that are personally important [65]. Following this principle, individuals make progress towards their goals by selecting and monitoring their behaviour over time [66]. In contrast, HBM also hypothesizes that fever is not sufficient a cue to action to make respondents proceed as for dengue. The uncertainty of the model’s conditions regarding the presence of fever increases the perception of susceptibility to dengue [67]. In a Venezuelan population, this explains a person’s intention to seek medical assistance as their first action if they suspect dengue infection, whereas treating at home would be their first choice in case of fever only [68]. Furthermore, most respondents prefer a healthcare facility that provides a higher level of medical attention than those offering basic services such as the barangay health stations, rural health units or private clinics which are readily available in the locality of respondents, reflecting the need to improve healthcare facilities in order to provide immediate and effective treatment to dengue patients. An interesting study in Cambodia reported a range of thought processes involved in the selection of healthcare facilities [69]. A lack of confidence over the quality of healthcare at the village level, suspicion as to the quality and provenance of locally available drugs, and real or perceived financial barriers to seeking care were predominant reasons for the sequence of treatment-seeking behaviours that was observed.

The systematic review also indicated no significant association between knowledge, attitude, and preventive practice regarding dengue was observed in Filipino populations. Although studies from Nepal [28], Indonesia [51], Vietnam [70] and Coimbatore, southern India [71] have each reported a positive association between KAP domains, other populations such as in Malaysia [72,73] and the Indian cities of Delhi and Kolkarta [74,75] have reported no correlation. An effective and sustainable strategy for combatting dengue is critically required when translating a community’s knowledge into good practices, such as the need to change their behaviour towards prevention of virus transmission. On account of this, carefully tailored practical approaches should be integrated into public health-related educational programmes, such as house-to-house inspections undertaken by healthcare personnel to conduct *Aedes* surveillance and to convey information and educate residents in a more personalised manner and familiar setting. Also, religious organisations, notably the predominant Roman Catholic Church in the Philippines, should be encouraged to influence and motivate habit change and to spur social mobilization, as is practised in other countries [76,77].

### Sources of information on dengue fever infection

This systematic review demonstrates that the principal sources by which information on dengue is disseminated to communities in the Philippines are television and radio. These outlets are key to delivering important knowledge regarding dengue, suggesting a need to maximize mass media in educating the population. A similar finding has been reported elsewhere [51,78,79]. The reason that television and radio are significant predictors of adequate knowledge of dengue could be that globally, and especially in developing countries, these traditional forms of audio-visual broadcast media remain the most popular means of communication that appeal to all age groups and to every socioeconomic class, encompassing both literate and illiterate members of the community. Thus, this finding indicates that television and radio should be fully utilized as an effective and accessible way to promote dengue awareness among Filipino communities. Surprisingly, it was also found that healthcare workers in the Philippines held a subsidiary role as dengue informants, in contrast to the data reported by studies conducted in Indonesia [51], Chitradurga in southwest India [80] and Malaysia [62]. The disparate findings may be due to patients’ perceived trustworthiness and acceptance of healthcare services. This could lead to behavioural impacts among the community such as the notable recent Dengvaxia® vaccine controversy experienced in the Philippines [81]. Given this possibility, dengue intervention programmes may need to be reviewed stringently to enable healthcare workers to maximize their educational impact on patients and their family members during clinic consultations, house visits or community outreach events.

### Dengue vaccination

The acceptance rates of dengue vaccination in this systematic review, ranging from 81.3 to 95.5% [40,42], are comparable to those reported by two studies conducted in Aceh Indonesia (70% and 77.3%, respectively) [82], one in Bandung, Indonesia (94.2%) [83] and another in Penang, Malaysia (88.4%) [84]. Although a higher level of education is associated with a better attitude towards dengue vaccine acceptance [85], a negative association has also been found [83]. Nonetheless, education is considered as an intermediate factor that could be affected by other considerations, which may explain the conflicting result as a predictor of dengue vaccine acceptance. Thus far, the association of income class and vaccine acceptance is not fully explained. In fact, it was proposed that wealthier people are more likely to comply with dengue vaccination primarily because they would consider the cost more affordable compared to people in a lower income class [83]. However, this systematic review showed opposite findings. Given that the majority of respondents included in the analysed studies were of lower income status and that more people were accepting of dengue vaccination, this might have tipped the scales appreciably towards significance.

Nonetheless, a previous study in Metro Manila, the Philippines, indicated a sufficiently high WTP for dengue vaccination, with mean WTP ranging between USD 27–32 [44]. This is greater than mean figures reported from comparative studies conducted in other countries; in Indonesia (USD 13.60) [83] and (USD 4.04) [85], in Nha Trang, Vietnam (USD 26.10) [86]; and in Medellin, Colombia (USD 22.60) [87]. On the contrary, the WTP for dengue vaccination of consumers was marginally higher in Brazil (USD 33.61) [88] and markedly so in Thailand (USD 69.80) [87]. Interestingly, a Vietnamese study of dengue patients with a history of hospital admission (for any ailment, not necessarily dengue) showed their elevated WTP for a vaccine (USD 67.40) [86]. This is probably due to an inflated awareness of the escalation of dengue cases due to time spent on hospital wards combined with the occurrence of a large-scale dengue outbreak in southeast Asia at the time reiterating the potential health benefits of vaccination.

### Complementary and alternative approaches to dengue control and prevention

In the Philippines the use of *E*. *hirta* to treat dengue exemplifies the importance of traditional medicine, particularly of herbal origin, to rural and remote communities lacking adequate vector control and with limited access to modern healthcare facilities, as reported previously [89,90]. Interestingly, the utilisation of herbal plants among community residents of Lugait in the Philippines has been reportedly endorsed by its local healthcare centres [32]. Similarly, in a study in the US, 53.10% of healthcare providers recommended at least one CAM to their patients [91]. This is in contrast to the perspectives of healthcare providers of using CAM as an adjunct to allopathic medicine in American Samoa [92] and Sierra Leone [93].

A recent systematic review of available scientific evidence reported the potential of *E*. *hirta* against dengue as it holds significant antiviral and platelet-increasing activities [94]. These conclusions may have been drawn due to this plant’s high concentration of reducing polyphenols as an active ingredient [95]. However, the mechanism of antimicrobial action remains to be determined, and the antiviral properties and its ability to stimulate blood platelet production are both currently under investigation [96]. Therefore, well-controlled clinical trials as well as contemporary pharmacological approaches, including activity-guided fractionation and elucidation of the mode of action in increasing platelet activity, are warranted to establish the potential use of *E*. *hirta* in a clinical setting.

### Lack of evidence on questionnaire reliability and validity

This systematic review demonstrates a clear need to determine the psychometric properties of the questionnaires used in dengue surveys conducted in the Philippines in order that KAP assessments are reliable, and the results are valid. A KAP study is a focused evaluation that measure changes in human knowledge, attitudes and practices in response to a specific intervention. As such, it is a quantitative research method that has the power to reveal a wealth of useful information on a significant aspect of research investigation. Therefore, if the questionnaire is well-constructed and the survey conducted by trained operators, a KAP study should assist in obtaining relevant data in a highly reliable and valid manner [97]. Reliability and validity are extremely important qualities required in order to measure the accuracy and consistency of this and other survey tools [98].

The survey questions should provide reproducible results (reliability test) and be assessed in three major forms of reliability: test-retest; alternate form; and internal consistency [99]. An Rs of value 0.70 or greater is generally accepted and indicates good reliability [100]. Despite the need to determine reliability during pretesting only a small minority of studies have reported Cronbach’s alpha coefficients during the pilot study and thereby confirmed the adequacy of internal consistencies of these scales [29].

In regard to validity there are several subtypes, namely face, content, criterion and construct validity [101]. The underlying construct of the items should be analysed by factor analysis to predict the discriminant and convergent validity [29,102]. In this systematic review, the majority of articles reported neither the results of a pilot study nor those of a pretest questionnaire–if these were indeed actually undertaken. Reliability, content and construct validity of a KAP structured questionnaire should be carefully examined. It is crucial to harmonize and validate the content of all the surveys with the aim of reducing the variability of findings based on questionnaires used for data collection.

### Strength and limitation

Several limitations of this review including inaccessibility of the original questionnaires which may have resulted in the heterogeneity of the findings in this review. Additionally, this could have resulted from the differences in statistical analysis or sociodemographic characteristics of the populations under study.

## Conclusions

This systematic review demonstrates a good level of knowledge, attitude and preventive practice regarding dengue among the resident population of the Philippines, particularly in highly endemic areas. Moreover, there is no association between KAP domains. Therefore, there is a great need to prioritize public health campaigns to target identified factors based on HBM. This is in order to raise the level of knowledge of dengue, to influence attitudes towards vector control and prevention and thereby to increase the uptake of preventive practices. These goals can be achieved through the active participation of communities and engagement with healthcare personnel, in combination with promotion of dengue awareness and safe complementary medicines through the use of television and radio. Equally important, there is an urgent need to determine the psychometric properties of KAP questionnaires before use in future dengue surveys in the Philippines in order for such assessments to be valid and conducted reliably.

## Supporting information

S1 Appendix PRISMA 2009 checklist. (DOCX)

S2 Appendix Review protocol. (DOCX)

S3 Appendix CASP checklist. (DOCX)

S4 Appendix Risk of bias assessment. (DOCX)
