# Supplementary material for: Different domains of dengue research in the Philippines: A systematic review and meta-analysis of questionnaire-based studies
Source: PLoS One. 2021 Dec 20;16(12):e0261412. doi: 10.1371/journal.pone.0261412 (PMC8687574; doi:10.1371/journal.pone.0261412)
Supplement: S2 Appendix — (DOCX) [file pone.0261412.s002.docx]

**Review Protocol**

**Background**

For questionnaire-based research, different domains are used to measure dengue in the Philippines, such as knowledge, attitude and preventive practices (KAP), dengue vaccine acceptance, the health belief model (HBM) association with dengue, and complementary and alternative medicines (CAM) to treat dengue. Far-reaching conclusions have been drawn from questionnaire surveys conducted in other endemic countries, providing a useful guide to decision makers in setting health policy priorities [1], assessing dissemination, application and cost-effectiveness of current guidelines, and closing important gaps in our knowledge of patterns of dengue transmission [2]. Studies have suggested that a combination of multidisciplinary and bottom-up approaches is more likely to be successful and sustainable way to combat dengue [3]. Prevention and control should be promoted in school and university curricula, as should the crucial role of healthcare volunteers in implementing effective social networks to raise dengue awareness of householders that may influence their attitudes and behaviour towards dengue [4]. Despite this, there has been a limited number of questionnaire-based studies in the Philippines compared to neighbouring countries, such as Malaysia, Thailand, and Indonesia.

Furthermore, the collective scopes have not been discussed previously in the context of researching a pattern for guidance. In addition, the accuracy of findings from questionnaire-based studies is a matter of concern, as the accuracy of results depends largely on the reliability of the questionnaires used in the survey [5]. A comprehensive review of questionnaire-based dengue-related studies is required to highlight the findings from all relevant previously published work, to assess the validity and reliability of questionnaires used in such research, as well as to draw broad conclusions. Thus, this systematic review and meta-analysis aims to summarize existing questionnaire-based studies conducted in the Philippines, which may help to improve survey design relating to different domains of dengue infection. In addition, it highlights future research needs and serves as a valuable reference for policymaking or health interventions focusing on the Filipino population.

**Review questions**

This review seeks to establish, through the available literature, what is best practice for smoking cessation. The specific review questions to be addressed are:

1. What are the collective scopes as well as gap of knowledge on questionnaire-based dengue-related studies that have been conducted in the Philippines?
2. How valid and reliable are the questionnaires used in the research?

**Inclusion criteria**

**Types of outcome measure**

The outcome of interest is include awareness/knowledge, attitude, preventive practice, treatment-seeking behaviour, sociodemographic variables and KAP, sources of information, vaccination, complementary/alternative dengue prevention, and the reliability and validity of questionnaire used in dengue survey.

**Types of studies**

The review considered all questionnaire-based studies evaluating KAP, dengue vaccine acceptance, the health belief model (HBM) association with dengue, and complementary and alternative medicines (CAM) to treat dengue.

**Search strategy**

The search strategy will be designed to access published materials and will comprise three stages:

(1) A search on various major electronic biomedical databases, such as PubMed, EMBASE, MEDLINE and ScienceDirect will be conducted to identify relevant keywords contained in the title, abstract and subject descriptors.

(2) Terms identified in this way, and the synonyms used by respective databases, will be used in an extensive search of the literature.

(3) Reference lists and bibliographies of the articles collected from those identified in stage two above will be searched.

The initial search terms will be ‘dengue’, ‘KAP’, ‘questionnaire’ and ‘Philippines’. Articles published within the period of January 2000 – April 2020 in English will be searched:

Full copies of articles identified by the search, and considered to meet the inclusion criteria, based on their title, abstract and subject descriptors, will be obtained for data synthesis. Articles identified through reference list and bibliographic searches will also be considered for data collection based on their title. Two reviewers will independently select articles against the inclusion criteria. Discrepancies in reviewer selections will be resolved at a meeting between reviewers prior to selected articles being retrieved.

**Data collection and critical appraisal**

Identified studies that meet the publication criteria will be grouped into one of the following categories: experimental studies, cohort studies, case control studies, interpretive and critical studies, cost minimisation studies and textual/opinion papers. The decision whether or not to include each article was made after reaching a consensus among the research team following group discussion between members via email. These studies will then be critically appraised by based on the eight critical appraisals of CASP Checklist [6]. Any disagreements that arise between the reviewers will be resolved through discussion and with the assistance of a third reviewer where required.

**Data synthesis**

A quantitative synthesis to derive meta-estimates of knowledge, perception and attitude of the study population and qualitative synthesis to describe the study population, study design, sampling methodology and outcomes will be conducted in the paper. For each study, primary outcome (knowledge, attitude and practice score) and secondary outcome (percentage of population with good knowledge, acceptable attitude, and practice) will be extracted. Knowledge, attitude and practice score were standardized to cent percent and pooled estimates are presented as mean and 95% confidence interval. Prevalence of population with good knowledge, acceptable attitude and practice were also identified, meta-analysed and presented also as mean and 95% confidence interval. Forest plots will be used to display pooled estimates. Heterogeneity will be tested using likelihood ratio test. Analyses will be performed using STATA 16 statistical software. For meta-analysis interpretation, a cut-off values for standardized knowledge score will be based on the following criteria; <64=poor, 64-80= Good ; >80= very good, based on previous study [7,8].

**References**

1. Lee JS, Mogasale V, Lim JK, Carabali M, Lee KS, Sirivichayakul C et al. A multi-country study of the economic burden of dengue fever based on patient-specific field surveys in Burkina Faso, Kenya, and Cambodia. PLoS neglected tropical diseases. 2019; 13(2): e0007164.

2. Marti R, Li Z, Catry T, Roux E, Mangeas M, Handschumacher P et al. A mapping review on urban landscape factors of dengue retrieved from earth observation data, GIS techniques, and survey questionnaires. Remote Sensing. 2020; 12(6): 932.

3. Selvarajoo S, Liew JWK, Tan W, Lim XY, Refai WF, Zaki RA et al. Knowledge, attitude and practice on dengue prevention and dengue seroprevalence in a dengue hotspot in Malaysia: A cross-sectional study. Scientific reports. 2020; 10(1): 1-13.

4. Dhimal M, Aryal KK, Dhimal ML, Gautam I, Singh SP, Bhusal CL et al. Knowledge, attitude and practice regarding dengue fever among the healthy population of highland and lowland communities in central Nepal. PLoS One. 2014; 9(7): e102028.

5. Taherdoost H. Validity and reliability of the research instrument; how to test the validation of a questionnaire/survey in a research. How to test the validation of a questionnaire/survey in a research. 2016.

6. Zeng, X.; Zhang, Y.; Kwong, J.S.; Zhang, C.; Li, S.; Sun, F.; Du, L. The methodological quality assessment tools for preclinical and clinical studies, systematic review and meta‐analysis, and clinical practice guideline: A systematic review. J. Evid. Based Med. 2015, 8, 2–10.

7. Selvarajoo S, Liew JWK, Tan W, Lim XY, Refai WF, Zaki RA et al. Knowledge, attitude and practice on dengue prevention and dengue seroprevalence in a dengue hotspot in Malaysia: A cross-sectional study. Scientific reports. 2020; 10(1): 1-13.

8. Yboa BC, Labrague LJ. Dengue knowledge and preventive practices among rural residents in Samar province, Philippines. American Journal of Public Health Research. 2013; 1(2): 47-52.
