# Supplementary material for: Different domains of dengue research in the Philippines: A systematic review and meta-analysis of questionnaire-based studies
Source: PLoS One. 2021 Dec 20;16(12):e0261412. doi: 10.1371/journal.pone.0261412 (PMC8687574; doi:10.1371/journal.pone.0261412)
Supplement: S4 Appendix — (DOCX) [file pone.0261412.s004.docx]

| **Paper for appraisal and reference** | **Section A: Are the results valid?** | | | | | | **Section B: What are the results?** | | | **Section C: Will the results help locally?** |
| --- | --- | --- | --- | --- | --- | --- | --- | --- | --- | --- |
|  | Was there a clear  statement of the aims of  the research? | Is qualitative  methodology  appropriate? | Was the research  design appropriate to  address the aims of the  research? | Was the recruitment  strategy appropriate to  the aims of the  research? | Was the data collected in  a way that addressed the  research issue? | Has the relationship  between researcher and  participants been  adequately considered? | Have ethical issues been  taken into consideration? | Was the data analysis  sufficiently rigorous? | Is there a clear statement of findings? | How valuable is the  research? |
| [25] | Y | Y | Y | Y | Y | Y | N | CT | Y | Educational influenced the implementation of control measures of dengue |
| [33] | Y | Y | Y | Y | Y | Y | N | CT | Y | Necessary plans to elevate moderate knowledge of dengue prevention and control |
| [34] | Y | Y | Y | Y | Y | Y | CT | CT | Y | Understanding of the influence of social and cultural aspects in dengue transmission in order to develop competent control and  prevention programs |
| [35] | Y | Y | Y | Y | Y | Y | Y | CT | Y | Urgent need to raise awareness and knowledge on dengue |
| [36] | Y | Y | Y | Y | Y | Y | N | CT | Y | Urgent need to raise awareness and knowledge on dengue |
| [37] | Y | Y | Y | Y | Y | Y | N | CT | Y | Need to employ effective behavioural strategies that will address ways to translate the people’s knowledge about dengue into positive practices |
| [38] | Y | Y | Y | Y | Y | Y | Y | CT | Y | Information from medias and from health centers enhances the awareness of other preventive measures to the community. |
| [39] | Y | Y | Y | Y | Y | Y | Y | Y | Y | Suitable program such as Communication for Behavioral Impact (COMBI) is a comprehensive strategy that uses communication of knowledge to have a significant effect upon behavioral change or increased practices against DF |
| [40] | Y | Y | Y | Y | Y | Y | N | Y | Y | Teachers play an important role in  facilitating of health promotion in dengue  endemic areas. |
| [41] | Y | Y | Y | Y | Y | Y | N | Y | Y | Reorientation training of community health workers should be conducted regularly to improve their technical skills and capability, and their ability to supervise prevention and control activities. |
| [42] | Y | Y | Y | Y | Y | Y | N | Y | Y | The most important factors associated with acceptance are educational attainment, employment status and income class. |
| [43] | Y | Y | Y | Y | Y | Y | N | Y | Y | Identifying barriers to action and to seek ways to translate population knowledge about dengue into positive preventive practices are deemed essential. |
| [44] | Y | Y | Y | Y | Y | Y | CT | Y | Y | Household size, knowledge regarding dengue, and attitude towards vaccination were significantly associated with willingness. |
| [45] | Y | Y | Y | Y | Y | Y | N | Y | Y | Mass vaccination campaign program in which part of the financial costs are covered by vaccine user charges is viable for the lower income groups |
| [46] | Y | Y | Y | Y | Y | Y | N | Y | Y | High FL values were obtained for symptoms observed during the febrile phase. |

**Note: Y=Yes; N=No; CT=Can’t tell**
